# Prevalence of 35de1G/GJB2 and del (GJB6-D13S1830) mutations in patients with non-syndromic deafness from a population of Espírito Santo - Brazil

**DOI:** 10.1590/S1808-86942010000400004

**Published:** 2015-10-19

**Authors:** Melissa de Freitas Cordeiro-Silva, Andressa Barbosa, Marília Santiago, Mariana Provetti, Eliete Rabbi-Bortolini

**Affiliations:** a Master's degree, professor at the Sao Pedro Integrated Facultes (Faculdades Integradas São Pedro), AEV/FAESA and doctoral student in biotechnology, RENORBIO/UFES; b Graduated in biological sciences, researcher; c Graduated in biological sciences, researcher; d Graduated in biological sciences, researcher; e Doctoral degree, research and outreach coordinator at the Sao Pedro Integrated Facultes, AEV/FAESA. Sao Pedro Integrated Facultes (AEV/FAESA)

**Keywords:** dna mutational analysis, gene frequency, deafness

## Abstract

Mutations in GJB2 gene are the leading cause of deafness in autosomal recessive inheritance, and the 35delG mutation is the most common in many ethnic groups. Besides the 35delG mutation in homozygosis, the mutation is also found in compound heterozygosis, coupled with other mutations in genes GJB2 and GJB6.

**Aim:**

To determine the prevalence of 35delG/GJB2 and del (GJB6-D13S1830) mutations in patients with sensorineural hearing impairment in residents from the Espirito Santo state, Brazil.

**Materials and methods:**

77 unrelated individuals with moderate to profound sensorineural hearing loss were evaluated. The 35delG mutation was studied by PCR / RFLP; and the del (GJB6-D13S1830) mutation was screened by the technique of multiplex PCR.

**Results:**

88.3% had normal genotype for the studied mutations, 1.3% were compound heterozygotes, 3.9% homozygotic for the 35delG mutation, 6.5% heterozygotic for 35delG/GJB2. The frequency of 35delG/GJB2 and del (D13S1830/GJB6) alleles in the sample was 7.8% and 0.65%, respectively.

**Conclusion:**

The data confirmed the existence of the mutations studied in cases of sensorineural hearing loss in a population from Espírito Santo / Brazil. These findings reinforce the importance of genetic diagnosis, which can provide early treatment for children and genetic counseling for the affected families.

## INTRODUCTION

Deafness is the most frequent sensory deficit in human beings; its reported incidence worldwide ranges from 1:300 to 1:1,000 children.^1^, ^2^, ^3^ The frequency in Brazil is estimated at 4:1,000 births.^4^ The etiology is genetic in about half of the cases worldwide; this includes syndromic and non-syndromic forms. Non-syndromic deafness accounts for 60 to 70% of inherited deafness that involve over 100 different genes, as follows: autosomal dominant (DFNA), autosomal recessive (DFNB), X-linked (DFN), and mitochondrial inheritance,^5^ of which the autosomal recessive inherited pattern is the most common. In several populations, the most frequent cause of non-syndromic autosomal recessive deafness occurs because of an altered connexin 26 protein, a communicating gap junction protein encoded by the gene GJB2 (13q11-12) (OMIM 121011).^6^, ^7^, ^8^, ^9^, ^10^, ^11^, ^12^, ^13^

Connexins are transmembrane proteins that form cell surface cylindrical hexameric structures which bind to other connexin hexamers in adjacent cells to form intercellular communication channels.^14^^,^^15^ Connexin 26 may be associated with other connexins in the inner ear. Connexin 26 recycles potassium ions as part of a signal transduction mechanism in the inner ear.^16^

Mutations in three connexin encoding genes, GJB2 (Cx 26), GJB6 (Cx 30), and GJB3 (Cx 31) have been found to cause hearing loss.^15^^,^^16^

The 35delG mutation accounts for most of the mutant alleles (60-85%) in Caucasians, among the DFNB-causing GJB2 gene mutations described so far.^6^^,^^7^^,^^9^, ^10^, ^11^, ^12^, ^13^^,^^17^

The 35delG mutation is a deletion of a guanine base in a sequence of six guanines that extend from nucleotide positions 30 to 35 on the GJB2 gene encoding exon, resulting in a stop codon. This deletion causes the polypeptide to be synthesized incompletely; it contains 12 amino acids, rather than the usual 226 amino acids.^18^

Analyses of the GJB2 gene in patients with autosomal recessive inherited deafness, especially because of the 35delG mutation, have shown that about 10 to 50% presented only one mutant allele.^19^ Other studies have suggested that there may be another mutation on the encoding exon of the Cx 26 gene; or the del (GJB6-D13S1830) mutation may coexist with the Cx 30 gene in heterozygote individuals for the GJB2 gene.^20^, ^21^, ^22^

Environmental factors cause about 80% of hearing loss cases in Brazil; the remaining 20% supposedly are of inherited causes.^4^ A recent study in the state of Sao Paulo has shown that the 35delG mutation was the most frequent in their sample (12.4%); it was found in 23% of the family cases, and 6.2% of the sporadic cases. The second most frequent mutation that these authors found was the del (GJB6-D13S1830) deletion on the GJB6 gene, which was found in 1% of cases and always coexisted with the 35delG mutation on the GJB2 gene.^23^

Estimates of the prevalence of 35delG heterozygotes in several European countries range from 2 to 4% of the normal hearing population.^6^^,^^17^ A Brazilian study revealed that 1 in 51 Caucasians (1.9%) have the 35delG mutation, which is similar to most European populations.^24^ Another study of neonates in Sao Paulo state showed that the 35delG mutation was present in 1 of 103 neonates (0.97%).^25^

Finding the prevalence of mutations in Brazil may help implement expedited molecular diagnosis tests to inform physicians about therapy, along with genetic counseling for family members of patients. It is therefore essential to learn about the genetic diversity of deafness to design improved proposals for the molecular diagnosis of population groups.^26^

The purpose of this study was to estimate the prevalence of the 35delG and D13S1830 mutations of the GJB2 and GJB6 genes in a sample of patients from Espirito Santo (Southeast Brazil), with bilateral prelingual non-syndromic sensorineural hearing loss of unknown causes.

## MATERIALS AND METHODS

### Patients and samples

A cross-sectional cohort study included 77 unrelated hearing loss patients, of which 38 were male and 39 were female. The age of patients ranged from 1 to 52 years. All resided in the state of Espirito Santo.

Patients came from auditory oral schools and hearing loss patient support centers in several regions of Espirito Santo; a clinical history to identify the onset of hearing loss, the presence of other cases in their families, to confirm non-syndromic deafness, and to exclude ambient causes, such as prenatal infection (rubella, toxoplasmosis, herpes, and post-natal infection, especially bacterial meningitis and exposure to ototoxic drug use. An audiological evaluation, immittance testing, audiological post-evaluation, pure tone audiometry and/or behavioral audiometry, and otoacoustic emissions testing showed that all patients had bilateral moderate to profound sensorial hearing loss.

Patients or caretakers signed a free informed consent form and provided 03 ml of peripheral blood for laboratory testing. The institutional review board of the Integrated Healthcare Center (Centro Integrado de Aten-ção à Saude or CIAS) approved this study (protocol no. 121/2006). A commercial DNA-extraction kit (Puregene DNA Purification Kit - Gentra Systems) was used to extract genomic DNA according to the manufacturer's instructions.

### Screening for mutations

A primer pair (F: 5 TCT TTT CCA GAG CAA ACC GC 3 and R: 5 GCT GGT GGA GTG TTT GTT CAC ACC CGC 3) was used to amplify the genomic region at 65°C annealing temperature for analyzing the 35delG mutation on the GJB2 gene. The resulting 89 bp PCR product was digested with the BstNI restriction enzyme. In the absence of deletions, the enzyme yields two fragments (69 bp and 20 bp); if the deletion is present, the enzyme does not cut the DNA (89 bp). The digested fragments were analyzed in 8% polyacrylamide gel electrophoresis.^27^

The del (GJB6-D13S1830) mutation was tracked using the PCR multiplex technique with three primers (F: 5 TTT AGG GCA TGA TTG GGG TGA TTT – 3; R1: 5 CAC CAT GCG TAG CCT TAA CCA TTTT – 3; R2: 5' TCA TCG GGG GTG TCA ACA AACA – 3') at 62°C annealing temperature.^20^^,^^21^ The F and R1 primers were used to detect the deletion, and the primer R2 was used to find the normal allele which amplifies a fragment within the deleted region. Two PCR products are obtained when the three primers are applied together, which makes it possible to separate homozygote, heterozygote or normal homozygote individuals. The amplified product was analyzed in 1.5% agarose gel electrophoresis.

## RESULTS

We originally evaluated 308 subjects with hearing loss; 77 of these had no environmental causes for their clinical picture, and were included in the study (25% of cases).

The 77 subjects with idiopathic deafness underwent molecular screening for the 35delG mutation in the GJB2 gene and the del (D13S1830) mutation in the GJB6 gene. The 35delG mutation was found in homozygosis in three patients (3.9% of cases), which established the etiology in these subjects. The 35delG mutation was found in heterozygosis in five subjects (6.5% of cases), but the presence of a single allele did not explain the cause of deafness in these patients. Normal and heterozygotic patients for the 35delG mutation were also investigated for the del (GJB6-D13S1830) mutation; one among 74 subjects had this mutation (1.35% dos cases). The heterozygotic patient for the del (D13S1830/GJB6) mutation was also heterozygotic for the 35delG/GJB2 mutation, which defined the double genetic etiology for this patient. These mutations were not found in 68 patients; other related mutations to Cx26 and Cx30 may be associated with the clinical pictures of these patients. Table 1 presents the results.

**Table 1:** Results of genetic investigation in this study

Genotypes	No. of subjects/total sample
35delG/35delG	3/77 (3.9%)
35delG/ N	5/77 (6.5%)
35delG/del (GJB6-D13S1830)	1/77 (1.3%)
N/N	68/77 (88.3%)

N: Absence of the 35delG/GJB2 and del (GJB6-D13S1830) mutations.

The frequency of the 35delG/GJB2 mutant allele in the sample was 7.8%; the frequency of the del (D13S1830/ GJB6) mutant allele was 0.65%.

## DISCUSSION

The genetic heterogeneity of non-syndromic hearing loss makes its molecular diagnosis more complicated, given the number of mutations that have been described in dearness-related genes; additionally, the predominance of each varies significantly in different populations.

The 35delG mutation in the GJB2 gene is the main cause of genetic deafness in Caucasian populations; it may be found in homozygosis or compound heterozygosis (with other mutations in the GJB2 and GBJ6 genes).^4^^,^^7^ The del (GJB6/D13S1830) mutation is the second most frequent DFNB-related mutation in European, Jewish and Brazilian populations.^7^^,^^23^

The frequency of the 35delG/GJB2 mutant alleles may vary in different regions of the world, as follows: United States of America (1.0%); Australia (1.0%); Austria (1.7%); Turkey (1.8%); Portugal (2.2%); Spain (2.5%), France (2.7%), and Italy (2-4%).^28^^,^^13^^,^^29^^,^^30^^,^^17^^,^^31^

Because of widespread racial mixing in Brazil, the 35delG/GJB2 mutation is not rare. Three studies in the state of Sao Paulo have revealed mutant allele frequencies ranging from 0.97% to 2.24%.^22^^,^^25^^,^^32^

A survey in 10 cities in different regions of Brazil revealed the following frequencies for 35delG mutation: North region (2.1%); Southeast region (1.5%); South region (1.2%), and Northeast region (0.8%); these differences were not significant.^33^

In the present study, the 35delG/GJB2 mutation and the del (D13S1830/GJB6) mutation were investigated in patients with idiopathic deafness in the state of Espirito Santo, Brazil. Among 77 subjects, nine unrelated individuals had the 35delG/GJB2 mutation; of these, one had the mutation in compound heterozygosis with the D13S1830 mutation in the GJB6 gene. The frequencies of the 35delG/GJB2 and del (GJB6-D13S1830) mutant alleles in the sample were respectively 7.8% and 0.65%. Our data concur with other studies done in the Brazilian population (Table 2, Table 3) and with several other studies that have documented the incidence of GJB2 and GJB6 gene mutations in patients with non-syndromic prelingual deafness.^28^^,^^29^^,^^34^, ^35^, ^36^, ^37^, ^38^, ^39^, ^40^

**Table 2:** Incidence of genotypes in the present study and other published papers

Genotypes	Present study	Pfeilsticker et al. 2004 ^41^	Piatto et al. 2004 ^42^	Batissoco et al. 2009 ^23^
35delG/35delG	3/77 (3.9%)	2/75 (2.66%)	5/33 (15%)	22/300 (7.3%)
35delG/N	5/77 (7.8%)	2/75 (2.66%)	3/33 (9%)	12/300 (4%)
35delG/ del (GJB6-D13S1830)	1/77 (1.35%)	N.A	1/33 (3%)	3/300 (1%)

N: Absence of the 35delG/GJB2 and del (GJB6-D13S1830) mutations;

N.A: No analyzed

**Table 3:** Incidence of 35delG/GJB2 and del (GJB6-D13S1830) mutant alleles in the present study and other published papers

Genotypes	Present study	Pfeilsticker et al. 2004 ^41^	Piatto et al. 2004 ^42^	Batissoco et al. 2009 ^23^
35delG allele	7.8%	4%	21%	9.8%
del (GJB6-D13S1830) allele	0.65%	N.A	1.5%	1%

N.A: Not analyzed

Although molecular analysis of hearing loss is not frequent in developing countries, it is essential to investigate GJB2 and GJB6 gene mutations for public health and genetic counseling purposes. The proportion of hearing loss patients because of genetic causes tends to increase as a result of investments and improvements in healthcare systems in developing countries such as Brazil. Establishing the prevalence and the types of mutations that cause non-syndromic hearing loss in Brazil, as was done in this study, may help implement simple and specific models to detect the main mutations causing genetic deafness in this country. The molecular diagnosis leads to accurate genetic counseling for family members and makes it possible to provide early rehabilitation for the children in affected families.

## CONCLUSION

Our data confirmed the presence of the 35delG mutation in the GJB2 gene in cases of non-syndromic bilateral moderate to profound sensorineural hearing loss in Espirito Santo, Brazil, a result which concurs with other published findings. The del (GJB6-D13S1830) mutation was found in compound heterozygosis with the 35delG/ GJB2 mutation in one patient. These findings underline the importance of a genetic diagnosis that may clarify the etiology and provide early treatment for children and genetic counseling for their family members.
